# Perspectives on care and communication involving incurably ill Turkish and Moroccan patients, relatives and professionals: a systematic literature review

**DOI:** 10.1186/1472-684X-11-17

**Published:** 2012-09-18

**Authors:** Fuusje M de Graaff, Patriek Mistiaen, Walter LJM Devillé, Anneke L Francke

**Affiliations:** 1 Medical Anthropology & Sociology Unit, University of Amsterdam, Oudezijds Achterburgwal 185, 1012, DK, Amsterdam, the Netherlands; 2 MUTANT, Projects for innovation and diversity, The Hague, the Netherlands; 3 NIVEL (Netherlands Institute for Health Services Research), PB 1568, 3500, BN, Utrecht, the Netherlands; 4 VU University Medical centre (EMGO+), PB 7057, 1007, MB, Amsterdam, the Netherlands; 5 Pharos (Knowledge and advisory centre on refugees, migrants and health), PB 13318, 3507, LH, Utrecht, the Netherlands

**Keywords:** Palliative care, Communication, Immigrants, Ethnic minorities

## Abstract

**Background:**

Our aim was to obtain a clearer picture of the relevant care experiences and care perceptions of incurably ill Turkish and Moroccan patients, their relatives and professional care providers, as well as of communication and decision-making patterns at the end of life. The ultimate objective is to improve palliative care for Turkish and Moroccan immigrants in the Netherlands, by taking account of socio-cultural factors in the guidelines for palliative care.

**Methods:**

A systematic literature review was undertaken. The data sources were seventeen national and international literature databases, four Dutch journals dedicated to palliative care and 37 websites of relevant national and international organizations. All the references found were checked to see whether they met the structured inclusion criteria. Inclusion was limited to publications dealing with primary empirical research on the relationship between socio-cultural factors and the health or care situation of Turkish or Moroccan patients with an oncological or incurable disease. The selection was made by first reading the titles and abstracts and subsequently the full texts. The process of deciding which studies to include was carried out by two reviewers independently. A generic appraisal instrument was applied to assess the methodological quality.

**Results:**

Fifty-seven studies were found that reported findings for the countries of origin (mainly Turkey) and the immigrant host countries (mainly the Netherlands). The central themes were experiences and perceptions of family care, professional care, end-of-life care and communication. Family care is considered a duty, even when such care becomes a severe burden for the main female family caregiver in particular. Professional hospital care is preferred by many of the patients and relatives because they are looking for a cure and security. End-of-life care is strongly influenced by the continuing hope for recovery. Relatives are often quite influential in end-of-life decisions, such as the decision to withdraw or withhold treatments. The diagnosis, prognosis and end-of-life decisions are seldom discussed with the patient, and communication about pain and mental problems is often limited. Language barriers and the dominance of the family may exacerbate communication problems.

**Conclusions:**

This review confirms the view that family members of patients with a Turkish or Moroccan background have a central role in care, communication and decision making at the end of life. This, in combination with their continuing hope for the patient’s recovery may inhibit open communication between patients, relatives and professionals as partners in palliative care. This implies that organizations and professionals involved in palliative care should take patients’ socio-cultural characteristics into account and incorporate cultural sensitivity into care standards and care practices*.*

## Background

Palliative care has seen considerable development in Western countries in the past few decades, and the number of hospices and other institutions specializing in palliative care has been growing [1-3]. Initially palliative care was mainly associated with care for terminally ill cancer patients. In recent years however, palliative care has expanded, including patients with non-malignant, progressive and life-limiting conditions such as heart failure and COPD [4]. Furthermore, palliative care providers and researchers increasingly pay attention to patients with specific cultural and socio-demographic characteristics, such as patients with a non-Western background. This is illustrated by the fact that in 2002 the World Health Organization clearly stated that guidelines on palliative care in all countries have to be adapted to cultural contexts [5].

Studies of palliative care conducted in non-Western countries have pointed to patients’ limited choices with regard to obtaining adequate pain relief and medication because of poverty [6-8]. Family care systems, religious practices and traditional care perceptions may influence the use of palliative care. Cultural minorities living in Western countries may face inadequate palliative care because of language differences [9], health literacy difficulties [10] or experiences of discrimination [11,12]. The supply of palliative care may not always meet the care expectations of immigrants due to their specific cultural or religious background [13-15]. Research among Sikh and Muslim patients with life-limiting illnesses living in Europe revealed that these immigrant patients are often reluctant to seek help from professional caregivers or institutions, rather than care from their own family and close relatives, because of negative experiences with care services (such as unacceptable food and racism) and concern about criticism from their community. Besides, communication with professionals was often hampered since illness and suffering were viewed as God’s will [16].

In our empirical research in the Netherlands over the period 2001 to 2009 we found care professionals often see specific care needs and communication problems when delivering palliative care to immigrant patients [17,18]. They often find it difficult to assess and meet the needs of these patients and their families, due to the patient’s lack of knowledge about the disease, cultural patterns within family relationships and inadequate formal or informal interpreter facilities. Turkish and Moroccan people form the largest immigrant groups in the Netherlands [19]. The ethnic roots of Turkish and Moroccan immigrants are diverse. However, most of them share important features such as coming from poor agricultural regions, arriving as low-paid ‘guest workers’ between 1965 and 1980, and living in deprived neighbourhoods as a Muslim minority. The first generations of Turkish and Moroccan immigrants are ageing now, and more and more of these people will start to need palliative care. An earlier literature study on the care needs of the Turkish and Moroccan elderly revealed that they often experienced barriers to making use of Dutch professional care, e.g. because of the strong role of family care in their cultures [20]. However, at that time we did not find any publications about Turkish and Moroccan people in the palliative phase. Since the number of patients in these groups needing palliative care will have increased since then, and accordingly the number of relevant studies can be expected to have grown, we decided to reinvestigate the international literature on Turkish and Moroccan incurably ill patients.

The questions addressed in this systematic literature review are:

What is known from previous research about

a) the care experiences and care perceptions of incurably ill Turkish and Moroccan patients, their relatives and care professionals?

b) communication between these patients, relatives and care professionals regarding care and treatment in the palliative phase?

## Methods

A systematic review was performed in several steps to find research literature about care perceptions and communication in the care for Turkish or Moroccan incurably ill patients. In this review we used the term ‘incurably ill Turkish or Moroccan patients’ to refer to people with a Turkish or Moroccan background, whether living in Turkey or Morocco or living in the Netherlands or another immigrant host country, who suffer from an incurable life-threatening disease. A person is defined as having a Turkish or Moroccan background if they were ‘born in Turkey or Morocco (with at least one parent born in Turkey or Morocco) or born outside Turkey or Morocco but with at least one parent born in Turkey or Morocco’.

### Searches

We searched for both qualitative and quantitative studies. Three main sources were used to find the literature: 17 national and international literature databases, four Dutch journals dedicated to palliative care and 37 websites for relevant national and international organizations. Additional file 1 gives the details of all sources. Additionally, members of the project team were asked for relevant publications and references listed in review articles were checked. The search string below was used for Pubmed. Searches for the other databases were derived from this Pubmed search string and adjusted where necessary (these are available on request).

"(“End-of-life” OR palliative OR hospice OR dying OR death OR "Advance Care Planning"[Mesh] OR "Hospice Care"[Mesh] OR "Palliative Care"[Mesh] OR "Withholding Treatment"[Mesh] OR "Terminal Care"[Mesh] OR "Euthanasia"[Mesh] OR “palliative sedation” OR “truth telling“ OR “truth disclosure” OR “advance directives”) AND (culture specific* OR culturally specific* OR diversity specific* OR culture sensitive* OR culturally sensitive* OR diversity sensitive* OR culturally divers* OR cultural aspect* OR cultural competen* OR “cultural context” OR racial OR etnic minorit* OR etnic specific* OR ethnic minorit* OR ethnic specific* OR “ethnic background” OR “etnic background” OR cross?cultural OR crosscultural OR trans?cultural OR transcultural OR intercultural OR inter?cultural OR multicultural OR multi?cultural OR indigeneous OR indiginous OR immigrant* OR migrant* OR ethnicity OR acculturation OR islam* OR Muslim* OR Hindu* OR Winti OR (Moroc* [tiab] OR Maroc* [tiab] OR Turk* [tiab] OR Surinam* [tiab] OR Antill* [tiab] OR aruba* [tiab] OR caribb* [tiab])) AND ("2000"[PDAT] : "2010"[PDAT])"

Manual searches were performed in specific journals (see Additional file 1). Relevant websites were searched without a predefined search strategy. Searches were limited to publications dating from 2000 onwards, since we found little relevant literature on the topic in our earlier literature study [20]. No language restrictions were applied. Although the searches were initially somewhat broader and aimed at finding literature on five immigrant groups in the Netherlands (see the search string above), it appeared that very few publications could be traced about the other three immigrant groups, so we restricted the inclusion criteria further to just Turkish and Moroccan people for the purpose of this paper.

### Inclusion and exclusion criteria

All the references obtained in this way were then checked to see whether they met the inclusion criteria. This was done first on the basis of the title/abstract and later on the basis of the full text of the documents. The inclusion process was carried out by two reviewers independently and disagreements were solved by discussion.

The inclusion criteria applied were as follows:

It is a primary empirical research paper.

The publication is about socio-cultural factors concerning Turkish or Moroccans subjects. Socio-cultural factors are factors that might affect the feelings and behaviours of groups with regard to care values and care practices, family and kinships structures as well as their attitude towards professional care. We included publications on Turkish and Moroccan patients in their countries of origin in addition to publications on Turkish and Moroccan immigrants in host countries.

A relationship between a socio-cultural factor and health (or care) outcomes was studied.

The paper concerns patients and/or carers of patients with an oncologic or incurable disease and/or in the palliative phase.

**Figure 1:**
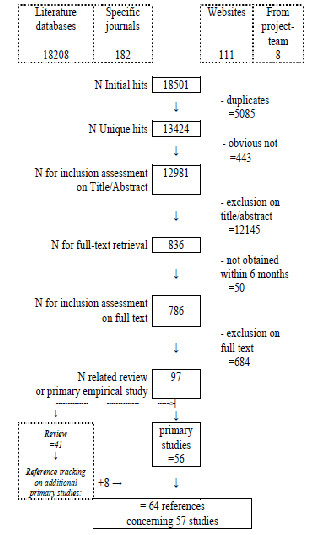
Flow diagram of the study’s selection process.

Exclusion criteria were letters/editorials, publications on organ donation, perinatology, near-death experiences, death row or mourning. All searches were done in May 2010. The inclusion flow diagram is depicted in Figure 1.

### Appraisal of the methodological quality

The methodological quality of the studies satisfying the inclusion criteria was checked. Since publications using different types of research methods (both qualitative and quantitative) were included, a generic appraisal instrument was applied [21]. This was chosen because it was specifically developed to appraise publications of disparate kinds of data. It consists of nine items (abstract, background, method, sampling, data analysis, ethics, results, transferability and implications); each item is scored on a 4-point scale from very poor to good (total scores may vary from 9 to 36; scores less than or equal to 18 were considered as ‘poor methodological quality’, from 19 to 27 ‘moderate’ and above 27 ‘good quality’). The methodological assessment was done by one researcher [PM] and 50% of the references were also checked independently by a second reviewer [AF, WD] (in this case the mean of the two scores was computed). Reviewers were assigned in such a way that they never had to judge documents of which they were a co-author. The publications in the Turkish language were assessed by a native Turkish psychologist. A low methodological score was not used as an exclusion criterion, but studies with such a low score were checked if they presented results that contradicted the results in other studies.

### Data extraction, analysis and synthesis

Data were extracted from each included publication by one reviewer and checked by a second. Data were extracted about the type of research, nature and size of the research population (ethnic group, professionals/patients/relatives, disease), country where the research was done, research questions and results.

Data from the publications that related to the review questions were extracted and classified in themes, which were then discussed in the research team. We analysed and synthesized the data in various ways, looking for contrasts: between Turkish and Moroccan patients, between experiences in countries of origin and experiences in host countries, between different care belief issues, between issues concerning care in practice and communication issues, and between the different phases that patients go through in the course of their illness. We concluded that the relevant data could best be presented in using the themes shown in Tables 1, 2, 3 and 4. For each theme, data from different populations (Turkish/Moroccan/mixed group of immigrants) and perspectives (patients/relatives/professionals) were described and summarized. If possible, a distinction was made between the different immigrant groups and between data concerning the countries of origin and data concerning the immigrant host countries.

**Table 1:** Findings relating to perspectives on family care

**Study**	**Family care as a duty**	**Family care as an economic necessity**	**Family care as a burden**
*Findings concerning Turkish patients*			
(Aksoy, 2005) [28]	It was important for a traditional Turkish family to look after ill and old parents. Family care was a duty.	*-*	*-*
(De Meyere, 2004) [47]	-	Some women had financial problems, e.g. they could not pay for a breast prosthesis.	Women became depressed if they had no family, feared losing their husband, or inducing gossiping in community.
(Groen-van de Ven and Smits, 2009) 2009 [55]	Ideally elderly live with their eldest son, cared for by their daughter-in-law, but often other relatives (daughters) cared for parents. Professional care was valued as not good enough.	*-*	Family care involved a lot of tasks (preparing food, housekeeping, accompanying patients to doctors, and personal care). Relatives became overloaded when practices did not correspond with their norms.
(Oksuzoglu et al., 2006) [68]	The role of family and social factors was very great in Turkey.	*-*	*-*
(Van den Bosch, 2010) [77]	-	Financial situation limited self efficacy in Turkish elderly.	*-*
(Yerden, 2000) [80]	Turkish active elderly expected their children (eldest son and wife) to take care for them. Their sons felt obliged to do so, but hoped to share this task with all the family.	*-*	Turkish elderly were often cared for by their children, but some children moved to other towns to flee from the heavy duty.
(Yerden, 2004) [81]	Ideally elderly live with their son’s family, many don’t. Daughters (in law) mainly do caring. When elderly are active they suppose family will care, when they become bedridden family care turns out to be insufficient.	*-*	Children support elderly by shopping, cleaning, administration, cooking and personal care.
*Findings concerning Moroccan patients*			
(Errihani et al., 2005) [52]	92% of patients were supported by family members.	For many families monthly revenues were not enough to pay for treatments, only 15% were insured.	*-*
(Errihani et al., 2006) [50]	92% of patients were helped by their family.	Poverty and lack of medical insurance limit professional treatments.	*-*
*Findings regarding Turkish and Moroccan patients ( sometimes other immigrant patients as well)*			
(De Graaff and Francke, 2003a) [17]	‘You should care for sick’ was an ideal, but also a must, enforced by fear of gossip in the community. Male relatives often rejected help from outside family. Professional help was more accepted when women were active in decision-making process.	*-*	Even in large extended families daily physical care was often carried out by one female family member.
(De Graaff and Francke, 2009) [45]	-	*-*	Limited use of home care often led to high care burden for relatives and improper care.
(Meulenkamp et al., 2010) [65]	Many elderly expected their children to care for them and to prepare and offer food as soon as visitors arrive.	The financial situation of immigrant elderly was often bad. Many elderly depended on their children for administration and financial management.	*-*
(Koppenol et al., 2006) [59]	Patients preferred to be cared for by their kin. Talking about illness and the sorrow it brings was often not done in Turkish and Moroccan families.	*-*	*-*
(Korstanje, 2008) [60]	Relatives felt responsible for patients and neglected duties regarding their work or children.	*-*	Family care sometimes led to financial problems. Relatives felt overburdened, but not able to discuss this as family care is obviously expected in their culture.
(NOOM, 2009) [23]	Parents and children created a dilemma: the ideal of children taking care of parents prevented them from looking for other solutions for meeting care needs.	*-*	*-*
(VPTZ, 2008b) [26]	Family care was an obvious response to a new situation, since immigrants often had no experience with dying people and did not know the supporting facilities in Netherlands. Turkish elderly often lived with a son or daughter, Moroccan families sometimes brought a helper from Morocco.	*-*	*-*
(Yerden and Van Koutrike, 2007) [82]	Men felt responsible for organizing care, but physical care and housekeeping was done by women.	*-*	*-*

**Table 2:** Findings relating to perspectives on professional care

**Study**	**Preferences for hospital care**	**Barriers to use of professional care / Perspectives on quality of professional care**
*Findings concerning Turkish patients*		
(Aksoy, 2005) [28]	General public: 47% would not send relatives to hospices, as it did not fit with traditional views on family duties; 54% would prefer to stay at hospital in their terminal phase.	*-*
	Health professionals: 55% would not wish relatives to stay in a hospice; 64% would prefer to stay at home in terminal phase, since not much can be done in hospitals for patients in their terminal phase.	
(Beji et al., 2005) [32]	In cases where treatment fails 43% of gynaecological cancer patients would prefer inpatient care, 41% outpatient care, 16,2% wished to go home. Main reason was feelings of security (90%).	*-*
(Betke, 2005) [33]	-	Turkish elderly had little experience with and knowledge about palliative care. They would like to have Turkish-speaking personnel, flexible \visiting hours, separate wards for male and female patients, no pork meat and accommodation for Muslim burial rituals.
(Cobanoglu and Algier, 2004) [40]	-	Physicians’ most mentioned problem in category of ‘social problems’ was limited resources of hospitals (49%).
(De Meyere, 2004) [47]	-	Groups for mutual help among migrant women were not effective as women did not feel socially safe within these groups.
		Talking to doctors was more comfortable as they are medical experts, who vowed secrecy.
(Ersoy and Gundogmus, 2003) [54]	Most GPs preferred to hospitalize patients, even by using force if necessary, in order to keep them from harm.	*-*
	Physicians were more often inclined to fulfil wishes of patients’ family, than to respect patients’ wishes, living will or previous consent of patient.	
(Meric and Elcioglu, 2004) [64]	33% of nurses preferred patients dying at home, 45% preferred patients dying in special facilities in hospitals and 22% preferred wards exclusively for terminally ill people.	Teamwork within hospitals and communication between professionals and patient and relatives should be improved. 64% of nurses thought psychiatrists should be working in palliative care, 18% opted for a palliative team comprising doctors, nurses, psychiatrists and relatives; 18% opted for a team including dieticians and physiotherapists.
(Oflaz et al., 2010) [67]	-	27% of nurses experienced difficulties in dealing with problems like unrelieved pain and suffering of patients, and their own sadness and anxiety; 61% felt they experienced inadequacy and helplessness about pain management and treatment; 29% experienced difficulties with emotional distress, e.g. when patients or relatives denied their situation, were dissatisfied or refused treatment.
(Van den Bosch, 2010) [77]	-	Many Turkish elderly had language problems when visiting GP. All patients having home care, used care from a Turkish home care organization.
		66% had been visiting doctors in Turkey, but these visits were expensive.
		Most Turkish elderly preferred using care in Netherlands, for insurance reasons and because care seemed better in Netherlands.
(Yerden, 2000) [80]	-	Elderly were too ashamed to ask for professional care. They accepted use of GP and hospital, but not use of home care and old people’s homes. Children were less negative about care provisions, but realized it would induce gossiping.
(Yerden, 2004) [81]	-	Home care and old people’s homes were becoming more accepted, daughters (in law) were more willing to use provisions than sons.
*Findings concerning Moroccan patients*		
(McCarthy et al., 2004) [63]	-	Nurses and physicians working in oncology centres felt embarrassed by shortage of means and training in treatment of cancer-related pain.
(Errihani et al., 2005) [52]	-	Poverty and illiteracy were major obstacles for patient care. Alternative treatment were visiting marabouts, fqihs, using medical plants.
(Errihani et al., 2006) [50]	-	Poverty and lack of medical insurance limited effective treatments: 85% of patients had no medical insurance and 52% had an income below 1500 DH per month whereas cost of disease was >35000 DH in 85% of cases.
		Chemotherapy was difficult to accept (90%), radiotherapy (54%) and surgery (40%) were more accepted.
*Findings regarding Turkish and Moroccan patients ( sometimes also other immigrant patients)*		
(ACTIZ, 2009) [22]	-	Relatives advised Dutch providers of home care and elderly care not to strive for standard culture-related care programs but for an individual approach. They asked for support in dealing with loss of independence of their patient and facilities to organize culture-specific rituals.
(Buiting et al., 2008) [35]	Immigrant patients’ rates of death in hospitals was high. (59% for immigrants vs 32% for Dutch)	*-*
(De Graaff and Francke, 2003a) [17]	-	Asking for professional care is restricted by feelings of shame, not only personally (being vulnerable or naked) but also socially (adopting ‘Dutch’ care instead of clinging to own traditions).
(De Graaff and Francke, 2009) [45]	-	Few Turks and Moroccans were referred to home care, especially those who did not speak Dutch.
		According to GPs and nurses, main barrier to home care was communication problems.
(De Graaff et al., 2010a) [18]	-	The views of Turkish and Moroccan patients and relatives on ‘good care’ and ‘ good communication’ were often different from views of Dutch care providers.
(Meulenkamp et al., 2010) [65]	-	Elderly migrants were concerned about hygiene and body care; they wished to take a shower daily. Women preferred to be cared for by female care providers.
		Professional care homes should have enough room for visitors and religious duties; professionals who can speak Turkish/Moroccan.
(Koppenol et al., 2006) [59]	-	Many Turkish and Moroccan elderly didn’t understand aetiology of illness, they feared cancer as contagious, and fatal. Patients asked for care in acute situations, but being unaware of diagnosis, they felt cured as soon as they felt a little better.
(Korstanje, 2008) [60]	-	Even in hospital relatives felt responsible for wellbeing of patient, they were very alert to professionals’ activities. Discharge from hospital was too abrupt, decisions were not sufficiently communicated.
(NOOM, 2009) [23]	-	Use of professional care was often impeded by the community (insisting on family care) and administrative procedures.
		Turkish and Moroccan men generally would like to have body hair shaven, but they didn’t dare to ask care providers to help them. Bathing should be done with running water.
(VPTZ, 2008b) [26]	Most Turkish and Moroccan patients preferred to die at home, but being in hospital was preferred if one still hoped for cure, or wanted to unburden the family.	Relatives did not ask for professional help, they wanted to stay in charge, and feared financial debts.
(Yerden and Van Koutrike, 2007) [82]	-	Relatives felt professional care didn’t fit with traditions of hygiene, halal food, and help of people of same sex.

**Table 3:** Findings relating to perspectives on end-of-life care and decision making

**Study**	**Hope for cure and faith in Allah**	**Perspectives regarding end-of-life decisions: euthanasia or hastening death / withdrawing or withholding life-prolonging treatments/continuing to offer food and artificial nutrition**	**Involvement in end-of-life care or decision making**
*Findings concerning Turkish patients*			
(Akpinar et al., 2009) [27]	-	40% of Turkish nurses found withdrawing life-prolonging treatments justified when no medical benefit; 81% preferred continuing treatment for a dying child (50% until brain death), 19% preferred palliative care. In case a baby would survive with physical or mental impairment 41% left decision to family, if progressive irreversible illness 65% found family should decide.	*-*
		68% agreed that artificial nutrition should always be continued.	
(Balseven Odabasi and Ornek Buken, 2009) [31]	-	Elderly patients accepted life-prolonging proposals of physicians. Even when patients wanted to die, relatives wanted them to live*.*	Clinicians should accord a larger role to patients in end-of-life decisions. Discussing medical ethical issues openly is not easy in Turkey, leaving physicians alone in decision making.
			Physicians need training to handle communication about reanimating or passive euthanasia.
(Beji et al., 2005) [32]	After learning diagnosis 87% of patients with gynaecological cancer wanted survival and 50% recovery. If treatment should fail 75% would struggle, 27% would trust in God.	Abstaining from life-prolonging treatments only in patients suffering from bad family relations, pain or depression (37%), 63% wanted to survive. If treatment was to fail, 63% would ask for life prolongation, 36.8% would refuse that.	*-*
(Celik et al., 2009) [38]	-	*-*	82% of nurses provided an environment suitable for family to say goodbye to deceased patient, 5% allowed family to help care for deceased patient. Nurses did not apply special cultural or spiritual activities.
(Cobanoglu and Algier, 2004) [40]	-	Withdrawing or withholding life-prolonging treatments was problematic for 48% of physicians and 46% of nurses. Physicians found withdrawing more difficult than not initiating treatment. Nurses felt uncertainty in absence of written DNR orders.	*-*
(Cohen et al., 2006) [41]	-	Acceptance of euthanasia is low in Turkey. Weak religious belief is most closely associated with higher acceptance of euthanasia, as well asyoung age, being from non-manual social class, higher education, belief in national traditions and history.	*-*
(Erer et al., 2008) [49]	-	*-*	87% of cancer patients declared that knowing diagnosis is a patients’ right; 92% found physician should inform them about disease and choices of treatment, but 77% regarded it to be an obligation for physician to provide this information. 43% agreed that patients could refuse treatment. 79% wanted to take part in decisions.
(Ersoy and Gundogmus, 2003) [54]	-	84% of physicians would prolong life of a patient who had stated he did not want to live with aid of artificial respiration devices, but if his wife wanted him to live, only 13% would respect patient’s wishes.	*-*
(Ilkilic, 2008) [56]	-	In case of a relative who cannot live without mechanical support, Turkish parents wished to prolong life as long as possible, referring to religious duties. German physicians proposed stopping care when a situation is medically hopeless.	*-*
(Iyilikci et al., 2004) [57]	-	58% of anaesthesiologists would continue prolonging treatment when family wanted cessation, 85% would continue life support when family wanted continuation, 68% would continue minimal support in case of no likelihood of recovery.	66% of Turkish anaesthesiologists had given DNR orders, 14% discussed DNR with family, 1% with patient, 83% with colleagues and 2% with Ethics Committee. 10% preferred doctors to decide, 31% preferred to decide with patient, relatives and responsible physician, 58% preferred consensus between hospital administrators, Ethics Committee, patient, relatives and physician.
(Karadeniz et al., 2008) [58]	-	28% of health professionals found life support should be decreased when patient wanted euthanasia, 35% rejected this option. 27% agreed with statement that euthanasia should be legalized in all countries, 24% was undecided and 49% disagreed with this statement. 43% would not perform euthanasia if legalized. 42% agreed with statement that a patient should decide on his right to live. 19% was undecided and 41% disagreed with this statement. 11% agreed with statement that if a patient wanted euthanasia, nutrition should be stopped, 14% was undecided, 75% disagreed with this statement.	*-*
(Kumas et al., 2007) [61]	-	51% of ICU nurses stated they did not have enough knowledge about euthanasia.	*-*
		56% stated it was patient’s right, 24% would support patient’s request for active euthanasia, 39% would support passive euthanasia. 75% opposed active euthanasia, 73% opposed passive euthanasia. 81% thought legalization would be exploited, 77% said it would be exploited for inheritance reasons. 40% thought passive euthanasia is practiced in some cases, 20% that it is never practiced in Turkey.	
(Mayda et al., 2005) [62]	-	84% of oncologists agreed euthanasia should be discussed in Turkey. 42% believed euthanasia is performed in Turkey. 38% advised incurable patients not to start therapy.	54% of oncologists thought patients should decide about euthanasia, 42% said families and doctors, 4% relied on families.
		34% had been asked for euthanasia. 44% did not object to euthanasia, 56% objected to it as unethical.	64% of those who performed euthanasia (n = 42) thought relatives and doctors should decide on withdrawing treatment in case of euthanasia.
		51% of oncologists had sometimes withdrawn treatment.	36% thought patients and doctors and patients should decide, 2% thought that only patient could decide stopping treatment.
(Oz, 2001) [69]	-	58% of nurses and physicians defined euthanasia as ‘allowing death, leaving patients to die’, 17% as ‘passive euthanasia, not active death determined by others’, 13% as ‘painless peaceful death’.	*-*
		21% of nurses and 18% of physicians had requests ‘to make death easy’. For 63% of nurses and 43% of physicians pain was major reason, but pain should be controlled.	
		For 14% of nurses and 30% of physicians loss of hope was major reason. Euthanasia seemed appropriate for 81% of nurses and 66% of physicians ‘if there was no possible treatment or other alternative’. 13% answered ‘if patient is conscious and he and his family wanted it’.	
		41% of nurses wanted an active role in euthanasia, 54% was undecided.	
		66% of nurses and 61% of physicians would not do it after legalization.	
(Ozdogan et al., 2004) [71]	-	*-*	Many physicians favoured a paternalistic approach and felt that patients are not able to cope emotionally with bad news.
(Ozkara et al., 2004) [73]	-	92% of physicians defined euthanasia as ‘the performance of death upon request of a patient, who has a progressive, unbearable and fatal disease after a long and painful period with no hope of recovery in today’s medicine, with assistance of a physician, in better conditions and without pain’.	*-*
		85% wished for a public debate. 61% did not approve of euthanasia. Arguments against euthanasia were: probable abuse (42%), ethically incorrect (25%), religious (19%) en illegal (11%).	
		30% of physicians felt practicing euthanasia should be punished; 56% thought that euthanasia is practiced,	
		19% had encountered a euthanasia request.	
(Pelin and Arda, 2000) [74]	-	62% of physicians supported euthanasia, 60% only on conscious and persistent demands, 33% under strict regulations, 32% only passive form. 39% did not support euthanasia, because doctors should save life (58%) and science might solve problems (54%).	Keeping information away from patients in disease process especially in poor prognosis was almost a common practice in Turkey.
(Tepehan et al., 2009) [75]	-	Nurses and doctors did not have same knowledge about different forms of euthanasia (active, passive, physician-assisted suicide and involuntary euthanasia); most well known was passive euthanasia (73% of doctors and 63% of nurses).	*-*
		56% of doctors and 53% of nurses wanted euthanasia to be legalized.	
		57% of nurses and 59% of doctors thought it is performed secretly.	
		40% of ICU doctors discontinued treatment in patients with an incurable disease on more than one occasion.	
		In ICU higher scores. If legalized 28% of ICU doctors would apply it versus 15% of paediatrics. 24% of ICU nurses would participate. Justifications for objecting to legalization were: not ethical, can be abused, religious beliefs and personal values. 65% of nurses and 73% of physicians agreed that individuals should have right to decide about own death.	
(Turla et al., 2006) [76]	-	Euthanasia is defined as the killing, through either an active or passive way, of someone who suffers from an illness which arouses pity and who will never get better when asked by either the person himself/herself or his/her relatives. 66% of health professionals thought euthanasia as such should not be performed. They were all worried about abuse of euthanasia, 30% noted religious reasons, 30% found it unethical and 15% illegal.	57% found it useful to have discussions about euthanasia.
			63% thought decision should be made by both physician and family.
		8% had been asked to perform euthanasia.	
		If it were legal in Turkey 74% would not perform it.	
		81% found euthanasia can be misused if it is legal.	
(Yaguchi et al., 2005) [79]	ICU physicians in Turkey preferred oral DNRe orders, while 80% of Western physicians would apply written DNR orders.	Withdrawing or withholding life-prolonging treatments: In case of patient in vegetative state, IC physicians in Turkey chose life prolonging more often than physicians in other countries.	*-*
		They would, like their colleagues of southern Europe, treat complications with antibiotics, colleagues in northern and central Europe would actively withdraw therapy.	
*Findings regarding Moroccan patients*			
(McCarthy et al., 2004) [63]	-	Use of oxygen + sedation stopped after complications. Morphine was used only for terminally ill patients.	*-*
(Errihani et al., 2005) [52]	48% of cancer patients were active believers. Faith helps control fear and incertitude.	*-*	*-*
(Errihani et al., 2006) [50]	After diagnosis of cancer 50% of practicing Muslim patients felt culpable and 93% started practicing. In both groups new behaviours: wearing of “hijab”, eating plants recommended in de Koran.	*-*	*-*
(Errihani et al., 2008) [51]	Those who don’t actively practice their belief (49%) felt guilty, while those who were practicing believed Allah was testing them, so they accepted disease.	*-*	*-*
*Findings regarding Turkish and Moroccan patients ( sometimes other immigrant patients as well)*			
(ACTIZ, 2009) [22]	Relatives thought Allah would decide on end of life.	*-*	Relatives noted that decision making within families was often difficult as it was not clear who had to decide on what topics.
(Buiting et al., 2008) [35]	Physicians’ data on treatment of immigrant and Dutch patients:	Immigrants had fewer euthanasia requests (3% of immigrants vs 25% of Dutch), were more often seen as incompetent (60% of immigrants vs 45% of Dutch), more often physicians’ decisions (39% of immigrants vs 25% of Dutch).	*-*
	less wish to hasten end (5% of immigrants vs 17% of Dutch).	Also less withholding of life-prolonging treatments for immigrants than for native Dutch (12% vs. 15%); more withdrawing (20% of immigrants vs 12% of Dutch), more respiration (38% of immigrants vs 16% of Dutch), more cardiovascular medicines. (30% of immigrants vs 11% of Dutch), but less artificial nutrition and hydration (12% of immigrants vs 28% of Dutch).	
(De Graaff and Francke, 2003a) [17]	-	*-*	Many Turkish and Moroccan patients wished to die in home country, but return is hampered when they have few relatives there and more relatives in Netherlands.
(De Graaff and Francke, 2009) [45]	-	*-*	According to GPs and nurses, patients do not understand them. Non-satisfaction is often rooted in communication problems, Turkish and Moroccan patients need coaching by GP and nurses.
(De Graaff et al., 2010a) [18]	Some relatives saw wanting best possible treatment, keeping hope alive and not stopping feeding and giving curative treatment as a religious commandment.	Main concerns about ‘good care’ at end of life expressed by Turkish and Moroccan families were: maximum treatment and curative care till end of lives, never having hope taken away, devoted care by families, avoiding shameful situations, dying with a clear mind and being buried in own country. These wishes often conflicted with values of Dutch professionals, like improving quality of life, giving sufficient pain and symptom relief.	Relatives wished to decide as a family what information is given to patient. This wish sometimes conflicted with views of Dutch physicians.
		‘Good’ care implied not stopping feeding.	
(Koppenol et al., 2006) [59]	The immigrants’ way to deal with cancer is influenced by belief in supernatural forces. Cancer can be seen as a test or trial by Allah.	*-*	*-*
(Korstanje, 2008) [60]	Relatives felt morally supported by religion.	*-*	*-*
(Mostafa, 2009) [66]	Cancer was like Allah is testing you.	*-*	Talking about cancer was not done in Turkish or Moroccan community.
(NOOM, 2009) [23]	-	*-*	Elderly and children believed that aged people deserved rest, children had to look after them, and decision making should be left to children.
(Van Wijmen et al., 2010) [78]	-	*-*	Significantly less use of ADs among people who had:
			- children and a good relation with them, (15% versus 8%),
			- only elementary or basic vocational training (24% versus 12% and 8% for those with secondary school and higher),
			- a religious belief (16% versus 10%).
(VPTZ, 2008b) [26]	Reciting Koran verses gave peace to many Muslims. Dutch care professionals were not involved in rituals.	*-*	In last days many people were assisting in practical matters, but personal care was often done by one or two women only.
			Islamic women were often not involved in funeral.

**Table 4:** Findings relating to perspectives on communication

**Study**	**Communication about diagnosis**	**Communication about pain, sorrow and mental problems**	**Language barriers**	**Communication within family and within community**
*Findings concerning Turkish patients*				
(Aksoy, 2005) [28]	61% of general public and 89% of professionals wanted to know diagnosis, but 58% of general public and 71% of professionals preferred to hide it from relatives.	*-*	*-*	*-*
(Atesci et al., 2004) [29]	55% of cancer patients were unaware of diagnosis, 45% were aware; 68% guessed it from treatment process; 15% had been fully informed.	More psychiatric disorder in patients aware of diagnosis. Physicians may be reluctant to reveal diagnosis and prognosis, as cancer is regarded as a death sentence.	*-*	*-*
(Bagcivan et al., 2009) [30]	-	Many patients did not want to talk about pain as they feared dependency on analgesics and did not want to upset relatives.	*-*	*-*
(Balseven Odabasi and Ornek Buken, 2009) [31]	85% of patients found that if a patient is diagnosed with cancer he should be informed about diagnosis.	*-*	*-*	*-*
	68% of physicians argued they had to tell diagnosis first to patients before informing relatives.			
(Betke, 2005) [33]	Turkish elderly preferred not to talk about cancer or any other negative diagnosis to very ill patient. They didn’t want to reveal diagnosis to an incurable patient and preferred doctors to tell truth only to relatives.	*-*	*-*	*-*
(Bozcuk et al., 2002) [34]	44% of cancer patients not aware of diagnosis.			Predictors of good emotional functioning: gender (males were more often accompanied) and social functioning.
	44% of cancer patients informed about diagnosis.			
	There was no significant difference between scores on quality of life (QLQ-C30) between patients who knew and patients who did not know diagnosis.			
(Buken, 2003) [37]	63% of patients diagnosed with cancer not aware of diagnosis**.** Level of informing increased with chance of recovery and patients’ socio-economic and educational level. 52% of physicians were ‘protective’, preferred not to tell truth. Reasons for not informing: lack of legal foundation and control and difficulty of changing protective physicians’ attitude.	*-*	*-*	*-*
(Cetingoz et al., 2002) [39]	75% of the general Turkish population had satisfactory knowledge of cancer, 13% thought cancer is contagious, 87% thought cancer is fatal, but 40% also believed that half of all cancers can be cured.	*-*	*-*	*-*
	84% wanted to be informed of cancer diagnosis, but only 63% wanted relatives to be informed.			
	Presence of cancer in a relative did not influence results.			
(De Meyere, 2004) [47]	-	*-*	Less educated patients sometimes got to false information and believed that cancer is contagious.	*-*
(Demirsoy et al., 2008) [48]	16% of patients (mixed cancer and non-cancer patients) were not aware of diagnosis; 84% knew diagnosis, but 19% of these reported a different diagnosis than on chart.	*-*	*-*	*-*
	66% of patients wanted to be informed about diagnosis, 33% did not want this, 95% wanted to know effects of available treatments.			
	90% of nurses wanted to inform patients about diagnosis and prognosis, 75% reported that patients should be informed.			
(Erer et al., 2008) [49]	87% of patients (more types of cancers, 38% breast cancer) agreed that patients had right to be informed about diagnosis.	*-*	*-*	*-*
(Ersoy and Goz, 2001) [53]	76% of nurses would in case a breast cancer patient asked for her diagnosis, tell truth (some after consulting physician); 24% would prefer not to tell it.	*-*	*-*	*-*
(Ersoy and Gundogmus, 2003) [54]	53% of physicians would reveal diagnosis to a HIV patient, 8% would tell family but not patient, 18% would tell it to patient and his family together.	*-*	*-*	*-*
	44% of physicians would tell patient his diagnosis of lung cancer, 19% would first investigate patients’ competence, 12% would ask consent of family, 16% would not tell patient, in order not to harm him.			
(Groen-van de Ven and Smits, 2009) [55]	The Christian Turks in this study obviously cared for parents, encouraged by faith and by community. Professional care could only be accepted when a trustful relation with relatives had been created.	*-*	*-*	*-*
(Ilkilic, 2008) [56]	-	*-*	Language barriers impeded physicians in making joint decisions with Turkish patients. They often could not understand discussions of patients and interfering relatives.	*-*
(Meric and Elcioglu, 2004) [64]	57% of nurses noted that patients were not informed about diagnosis and prognosis.	29% of nurses faced problems in communication about care for terminally ill patients, they had feelings of empathy and loss when people die, 7% saw no problems and 63% had problems with some ‘special’ patients.	*-*	*-*
	58% thought patients should not be informed about prognosis.			
	All nurses felt relatives should be informed, 66% wanted to inform when diagnosis is clear, 34% wanted to inform when problems arise.			
(Oflaz et al., 2010) [67]	96% of nurses did not want to inform patient about his terminal status.	*-*	*-*	*-*
	60% did not think a person should be told his illness is incurable or terminal.			
(Oksuzoglu et al., 2006) [68]	48% of relatives wanted patient to be informed; 39% thought diagnosis should not be disclosed, 13% was hesitant.	*-*	*-*	*-*
	87% wanted family to be informed, 80% of the 39% that did not want to inform patient, wanted only family to be informed.			
	89% thought treating doctor should be messenger.			
(Ozcakir et al., 2008) [70]	Medical students found talking to patients important, patients should get permission to die at home. Students disagreed that incurably ill patients should be told diagnosis.	*-*	*-*	*-*
(Ozdogan et al., 2004) [71]	66% of relatives did not want patient to be informed about diagnosis**;** 57% because patient would be upset, 29% because patient would not want to know it.	*-*	*-*	*-*
(Ozdogan et al., 2006) [72]	The percentages that never, rarely, generally, and always told truth about diagnosis were 9%, 39%, 45% and 7%.	*-*	*-*	*-*
	54% of physicians felt influenced by requests from relatives, 46% not. Physicians who felt influenced told truth less often than those who did not. Training in breaking bad news resulted in higher scores of telling truth.			
(Pelin and Arda, 2000) [74]	93% of physicians thought patients should be informed about diagnosis, but one should avoid talking about life expectancy. 30% said they may choose to inform relatives.	*-*	*-*	*-*
	Older and more experienced physicians gave more information.			
(Van den Bosch, 2010) [77]	Many Turkish patients generally trusted medical competence of GP, but communication was limited as Turkish elderly did not accept idea of incurability and felt discriminated or not being taken seriously. Some thought GP was not informing them fully.	Many Turkish patients did not want to talk about psychological problems.	*-*	*-*
(Yerden, 2000) [80]	-	-	-	Communication within family about future needs and solutions was limited.
*Findings regarding Moroccan patients*				
(Errihani et al., 2005) [52]	33% of patients (diagnosed with cancer) were not aware of diagnosis and prognosis.	*-*	*-*	*-*
	In 89% of cases relatives were informed, in 9% only some very close kin, in 2% of cases patients lived with their disease in full discretion.			
	1% of patients’ families thought that cancer is contagious.			
(Errihani et al., 2006) [50]	-	*-*	Illiteracy and insufficient knowledge of Arabic caused problems. 37% were illiterate and 25% spoke Berber only.	*-*
(McCarthy et al., 2004) [63]	-	Physicians and nurses believed that pain in children with cancer is rarely expressed in Morocco, which hampered assessment of pain. For some Moroccans pain was inevitable. Some feared morphine addiction.	*-*	*-*
*Findings regarding Turkish and Moroccan patients ( sometimes other immigrant patients as well)*				
(De Graaff and Francke, 2003a) [17]	Several Turkish and Moroccan relatives said diagnosis and prognosis given by GP was not understood.	*-*	Important information came from relatives or members of community who worked in health sector.	Informing within community is hampered by fear of gossiping.
(De Graaff and Francke, 2009) [45]	GPs and nurses perceived a taboo on communication about diagnosis of terminal illnesses.	*-*	GPs and nurses found it difficult to distinguish patients’ wishes apart from relatives’ wishes, especially when relatives were translating.	GPs and nurses didn’t recognize influence of local migrant community on decision making in care.
(De Graaff et al., 2010a) [18]	*Communication about diagnosis*	*-*	In cases with language barriers Dutch professionals struggled with relatives who were acting as interpreter and as person in charge.	Relatives needed information about diseases and choices of treatments, they lacked role models in care for dying patients.
	Dutch professionals wished to inform patient and to realize advanced care planning, while Turkish and Moroccan relatives wished to keep patients’ hope alive. Turkish and Moroccan patients disliked direct way in which Dutch care providers informed patients.			
(Meulenkamp et al., 2010) [65]	-	*-*	Many elderly depended on children for administration and financial management. Some were illiterate; others did not have enough mastery of Dutch language.	Many migrant elderly enjoy participating in activities, but they also fear gossiping of compatriots.
(Koppenol et al., 2006) [59]	Relatives impeded doctors in informing patient about cancer diagnosis, although breast cancer women were often informed, because of surgery decisions. Many patients were in need of information, they felt bad, wanted to know reasons for complaints.	*-*	*-*	Some patients didn’t want other people to be informed about illness; they feared gossiping and social isolation.
(Korstanje, 2008) [60]	-	*-*	Relatives didn’t contact professionals because of language problems. Nurses were not well-informed as they spoke with Dutch-speaking relatives, who were not primary carers.	Caring relatives often felt overburdened, but could not discuss it, since helping was obviously expected.
(Mostafa, 2009) [66]	-	Most women (with breast cancer) felt GP did not listen, resulting in late diagnosis. Women wanted treatment for physical complaints, not for mental care.	*-*	*-*
(NOOM, 2009) [23]	Patients and relatives disliked direct way Dutch care providers informed patients about diagnosis.	Many immigrants didn’t talk about mental problems, instead they mentioned headache, pain in back or belly. Alzheimer disease was not recognized.	Assessment of symptoms was hampered by language barriers, as patients lost knowledge of Dutch.	Parents and children created a dilemma: ideal of children taking care of parents prevented them from discussing openly and looking for real solutions.
(VPTZ, 2008b) [26]	First generation immigrants did not talk about life-threatening illnesses. They were not amused by Dutch doctors informing them about diagnosis with little respect for family relations. Second generation immigrants wanted to be informed.	*-*	*-*	Relatives who cared for dying patients advised discussing illness and dying within Moroccan and Turkish communities.
(Yerden and Van Koutrike, 2007) [82]	-	*-*	Elderly faced language barriers, so children did administration.	*-*

## Results

### Characteristics of the studies included

Sixty-four publications [17,18,20,22-83] were included concerning 57 studies. Some studies were presented in more than one publication. In those cases we used the main publication for the description of the characteristics and study findings.

### Language and place of study

All studies were published in the period 2000 to 2010. Most of the 57 studies were published in English (39), one was in French [52] one in German [56], two were in Turkish [31,64], and fourteen in Dutch.

Forty studies addressed the experiences of Turkish subjects. Seven of these forty studies concerned immigrants with a Turkish background either living in Germany (one study), Belgium (one study) or the Netherlands (five studies). Of the 33 studies of Turkish people living in Turkey, five studies were performed in provinces in Eastern Turkey, but most concerned the situation in modern cities in Western or Central Turkey.

Four studies focused on Moroccans in Morocco and addressed the experiences of people living in Rabat and Casablanca, two large cities in Morocco with specialized cancer care.

Additionally, we found thirteen studies of Turkish and Moroccan immigrants in the Netherlands. Four of these thirteen studies focused on Turkish and Moroccan immigrants only, while the other nine presented data about several immigrant groups living in the Netherlands, including Moroccan and Turkish immigrants.

### Research questions and subjects

Most research questions addressed the needs or attitudes of care users or care providers, the problems of care professionals or relatives, or factors influencing the access to or the quality of care.

Patient perspectives were described in 22 of the 57 studies, relatives were involved in 15 studies, physicians in 23 studies and nurses in 17 studies. Some studies combined the perspectives of patients and relatives, for example when relatives were asked to describe the views of their ill patients [17,22,33]. Some studies combined the perspectives of relatives and professionals [23,26,59,66]. Other studies compared the views of patients and relatives with the views of professionals [18,45,48,69] or compared the views of physicians with the views of nurses [40,75].

### Designs, sample sizes and instruments

Many studies (35 of the 57) had a quantitative design, often using self-developed survey questionnaires. However, three studies [29,30,34] used existing instruments, namely the Hospital Anxiety and Depression Scale, the Barriers Questionnaire II (BQ-II) or the European Organisation for Research and Treatment of Cancer Quality of Life Questionnaire.

Twenty studies had a qualitative design, generally using semi-structured interviews or focus groups. Two studies used a mixed design. Studies performed in Turkey and Morocco were often quantitative in nature, involving large samples: e.g. 150–1,000 Turkish professionals or 600–1,600 Moroccan patients. Sample sizes in studies performed among Turkish or Moroccan immigrants in Europe were often smaller (less than 20 research subjects), related to the fact that these studies often had a qualitative design (see Table 5). One exception is the study by VPTZ (Vrijwilligers Palliatieve Terminale Zorg Nederland = Volunteers for palliative care in the Netherlands) describing focus groups with a total of 255 Moroccan and Turkish relatives.

**Table 5:** Methodological characteristics of the studies included

**Reference, language/ country [nr]**	Research question*	**Research subjects**	**Design**	Data collection (variables/topics measured)*	**Meth. score**
*Studies concerning Turkish patients*					
Akpinar et al., 2009, English/Turkey [27]	What are intensive care nurses’ attitudes to end-of-life decisions?	54 **nurses** at a Paedriatric Intensive Care Nursing Symposium and 101 nurses at a Congress on Intensive Care Nursing.	Quantitative study using questionnaires	Self-administered questionnaire on personal and professional characteristics, attitudes to end of life and futile treatment.	25.5
Aksoy, 2005, English/Turkey [28]	What are reasons for limited use of hospice facilities in Turkey?	200 **volunteers**, half of them were **nurses or physicians** in Ankara, Izmir , Sanliurfa or Erzurum.	Quantitative study using questionnaires	Self-administered questionnaire on preferences for staying in hospital or at home, for sending relatives to hospital or letting them stay at home, for knowing diagnosis and letting relatives know their diagnosis, and experiences with courses on terminal care.	16
Atesci et al., 2004, English/Turkey [29]	How is awareness of cancer diagnosis related to presence of psychiatric morbidity?	117 **patients** having chemotherapy in different departments.	Quantitative study using questionnaires and assessment by a psychiatrist	Hospital Anxiety and Depression Scale, General Health Questionnaire. Psychiatric diagnosis (DSM-IV) Added questions: What do you think you are suffering from? And Why do you think you are in hospital?	32
Bagcivan et al., 2009, English/Turkey [30]	What are barriers to pain management of cancer patients? What factors determine barriers in Turkish population?	170 oncology **outpatients** of Gulhane Military medical academy Hospital having been or still using an analgesic for cancer-related pain.	Quantitative study using questionnaires	BQ-II measures about beliefs concerning fear of addiction, fatalism, anxiety, not complaining,treatment, side-effects, immune system, analgesics masking symptoms. Some items were added from Brief Pain Inventory and from Pain Management Index.	35.5
Balseven Odabasi and Ornek Buken, 2009, Turkish/Turkey [31]	What ethical decisions are made in Turkey in diverse clinical situations and what is influence of patients, physicians and family members on decisions?	150 **outpatients** visiting Hecettepe University Hospital in 2007–2008 and 151 physicians (surgeons, pediatricians, anesthetists, internists).	Quantitative study using questionnaires and use of vignettes	Questionnaire and vignettes developed by Ruhnke et al. (2000), addressing whether patients should be informed about an incurable cancer and whether a terminally ill patient wishing to die should be ventilated.	33
Beji et al., 2005, English/Turkey [32]	What are reactions of gynaecologic cancer patients to poor prognosis and what are their preferences regarding location of terminal care and life-sustaining technology?	68 **patients** visiting gynaecologic-oncology policlinic of Istanbul University.	Qualitative study using semi-structured interviews	Self-administered topic list on concerns and reactions to diagnosis, desires for location of terminal care and preferences for withdrawing or withholding life-sustaining technologies.	25.5
Betke, 2005, Dutch/Netherlands [33]	What are specific needs of Turkish elderly regarding palliative care and how can Dutch nursing homes fulfil these needs?	Interviews: 10 Dutch and 2 Turkish informants, (**nurses, physicians** and others). Focus groups: 32 Turkish **elderly** who were not ill yet.	Qualitative study using semi-structured interviews and focus groups	Topics addressed in interviews and focus groups were: experiences with delivered care, information needs, wishes regarding accommodation, food and staff.	26.5
Bozcuk et al., 2002, English/Turkey [34]	What is current truth-telling practice for cancer patients in Turkey and does disclosure of truth affect quality of life?	100 cancer **patients**: 29 lung cancer, 23 breast cancer 5 colon cancer, 6 gastric cancer, 10 head and neck cancer, 26 other cancers.	Mixed-method design combining questionnaires with interviews	Interviews: items on family support. Questionnaire: EORTC QLQ-C-30 measuring five functional, nine symptom scales and global health status.	27
Buken, 2003, English/Turkey [37]	How can physicians give bad news to a terminally ill patient in an appropriate manner?	Questionnaire for 58 **physicians** and 150 medical students. Interviews with **82** newly diagnosed cancer **patients** about different services.	Mixed method design using questionnaires and interviews	Questionnaire and interviews about attitude of Turkish physicians, interferences with truth telling and regulations on information giving and patients’ rights.	17.5
Celik et al., 2009, English/Turkey [38]	What does a nurse do to care for deceased patients in ICU? What factors influence this care?	29 **nurses** working in cardiovascular urgical ICUs.	Qualitative observational study	Observations concerned nursing interventions for patient and emotional support for patients’ relatives.	25
Cetingoz et al., 2002, English/Turkey [39]	What is basic knowledge of and general attitudes about cancer?	630 subjects**,** none was known to have cancer **(general public)**.	Quantitative study using questionnaires	Self-administered questionnaire on knowledge about cancer (e.g. signs, symptoms, preventive measures, treatment modalities) and on attitude (e.g. desire to know diagnosis, to change lifestyles).	20
Cobanoglu and Algier, 2004, English/Turkey [40]	What are perceptions of physicians compared with perceptions of nurses on ethical problems in ICU?	21 **physicians** and 22 **nurses** working in Intensive Care Units in hospitals in 3 cities in Turkey.	Qualitative study using focus groups	Focus group topics concerned end-of-life decisions, communication, hierarchical and social problems. More specified categories were euthanasia, futile treatment, DNR decisions, autonomy as parts of end-of-life decisions.	32
Cohen et al., 2006, English/Turkey [41]	Is terminating life of incurably ill accepted in 33 European countries? What are associations with acceptance of euthanasia and social and religious factors?	Data of European Values Study of 1999–2000 including 1206 respondents in Turkey (**general public).**	Analysis of quantitative questionnaire data	European Values Study regards more than 300 items, e.g. one regarding acceptance of euthanasia.	34
de Meyere, 2004, Dutch/Belgium [47]	How do Turkish women in Belgium deal with breast cancer?	1 Moroccan, 3 Turkish and 7 Flemish informants **(physician, nurses and others).**	Qualitative study using semi structured interviews	Interview topics were social, medical and psychological problems around breast cancer.	22
Demirsoy et al., 2008, English/Turkey [48]	What are nurses’ and patients’ attitudes regarding information sharing about medical diagnosis and prognosis?	166 **nurses** and 435 **patients.**	Quantitative study using questionnaires	Self-administered questionnaire on informing patients with a limited life expectancy about diagnosis and prognosis, preserving hope and family support in informing patients.	25.5
Erer et al., 2008, English/Turkey [49]	What are views and expectations of cancer patients regarding information and autonomy?	104 **patients** attending outpatient clinic.	Quantitative study using questionnaires	Self-administered questionnaire on patients’ rights regarding being informed and autonomy.	30.5
Ersoy and Goz, 2001, English/Turkey [52]	Can nurses recognize ethical problems and how do they use ethical principles in decisions?	165 **nurses** working at bedside, encountering real ethical problems.	Quantitative study using questionnaires	Questionnaire on cases involving nursing care and ethical principles (autonomy, veracity, beneficence or justice).	26.5
Ersoy and Gundogmus, 2003, English/Turkey [54]	Can physicians recognize ethical problems and how do they use ethical principles in decisions?	207 **physicians** working in a hospital or primary health centre in Kocaeli.	Quantitative study using questionnaires	Questionnaire addressing cases of physicians involving care and ethical principles (autonomy, non-maleficence, beneficence or justice).	24
Groen-Van de Ven and Smits, 2009, Dutch/Netherlands [55]	What are experiences of family members of Suryoye elderly patients?	15 **Relatives.**	Qualitative study using semi-structured interviews	Topics were care history, experiences of giving care, personal caring qualities, influence of (Christian) religion and gender.	26
Ilkilic, 2008, German/Germany [56]	What conflicts occur in intercultural end-of-life care? What concepts and instruments can help German doctors and Turkish families to solve them?	3 cases of dying **patients.**	Qualitative design using case studies	Examination of thoughts about euthanasia, brain death, life prolonging measures, right to know your diagnoses, right not to know it, hierarchies in decision-making positions within family and potentials and limitations of living wills.	19
Iyilikci et al., 2004, English/Turkey [57]	What are practices of Turkish anesthesiologists with regard to withholding and withdrawing of life support from critically ill?	369 **anesthesiologists.**	Quantitative study using questionnaires	Self-administered questionnaire on education, religion, ICU facilities, bed capacity, ICU experience, experience with euthanasia, DNR orders and DNR decisions, and knowledge of Turkish Penal Code.	24
Karadeniz et al., 2008, English/Turkey [58]	What are attitudes of health professionals towards euthanasia?	510 health staff , viz. 309 **physicians**, 332 **nurses,** 91 **midwifes.**	Quantitative study using questionnaires	Questionnaire based on Euthanasia Attitude Scale: items on status of brain-dead persons, life-extending technology, ethics and legal issues.	29
Kumas et al., 2007, English/Turkey [61]	What are opinions about euthanasia of nurses who work in intensive care units?	186 **nurses** of 3 hospitals.	Quantitative study using questionnaires	Self-administered questionnaire on nurses’ knowledge about euthanasia, their definitions, their sources of information, euthanasia practices.	26
Mayda et al., 2005, English/Turkey [61]	What is attitude of oncologists towards euthanasia in Turkey?	85 **oncologists** participating in a scientific meeting.	Quantitative study using questionnaires	Self-administered questionnaire on physician’s approach to euthanasia, legal basis, euthanasia requests, actions towards passive euthanasia and expectations.	26.5
Meric and Elcioglu, 2004, Turkish/Turkey [64]	What problems face nurses in de communication with terminally ill patients?	125 **nurses** working on different disciplines/wards with < 10, with 11–50 and > 50 dying patients per year.	Quantitative study using questionnaires	Self-administered questionnaire on informing patients and relatives about prognosis, talking near unconscious patients, participation of psychiatrist and physiotherapists in palliative care team, feelings of attachment, empathy and loss, and recommendations to improve care.	36
Oflaz et al., 2010, English/Turkey [67]	What are concerns of nurses who care for oncology patients and how are these concerns related to their working experiences?	157 **nurses** of oncology units.	Quantitative study using questionnaires	Questionnaire is an adaption of list of feelings in Durham & Weiss (1997) on concerns for dying patients. Added items on care for terminally ill patients and pain treatment and management.	31
Oksuzoglu et al., 2006, English/Turkey [68]	What are attitudes and opinions of people accompanying cancer patients regarding cancer diagnosis disclosure?	270 **relatives** of patients visiting outpatient unit for chemotherapy.	Quantitative study using questionnaires	Self-administered questionnaire on telling patient and relatives about cancer diagnosis, on timing of telling truth and from whom it should be learned.	22
Oz, 2001, English/Turkey [69]	What are views on euthanasia of nurses and physicians working at a hospital in Ankara?	113 **nurses** and 84 physicians.	Quantitative study using questionnaires	Self-administered questionnaire on definitions of euthanasia , views on for whom it may be appropriate, methods, conditions, kinds of help and information, feelings towards patients requesting euthanasia, responsibilities, willingness to take a role now or when it becomes legal.	27
Ozcakir et al., 2008, English/Turkey [70]	What are attitudes and opinions of medical students about doctor-patient communication in case of dying patients and patients with chronic diseases?	253 first-year **medical students** of 2004–2005.	Quantitative study using questionnaires	Self-administered questionnaire on experiences in caring for someone with a serious disease and doctor-patient communication in case of patients with chronic diseases or dying patients.	19
Ozdogan et al., 2004, English/Turkey [71]	What are relatives’ attitudes towards informing cancer patients about their diagnosis? What factors affect this attitude?	**Relatives** of 150 patients visiting outpatient unit.	Quantitative study using questionnaires	Self-administered questionnaire on attitude towards informing patients with cancer, religious beliefs, daily activities, preference about disclosure, previous requests for information and previous knowledge about cancer.	27
Ozdogan et al., 2006, English/Turkey [72]	What are self-reported truth-telling practices of physicians and what factors influence these in Turkey?	131 **physicians** participating in 15^th^ National Oncology Meeting in 2003.	Quantitative study using questionnaires	Self-administered questionnaire on attitude towards informing patients, views on patients’ mood, knowledge, wishes to be informed about diagnosis.	26.5
Ozkara et al., 2004, English/Turkey [73]	Do physicians approve of legalization of euthanasia and assisted suicide? Is this correlated to their specialties, sex and workplace?	949 **physicians** working in 7 different areas in Turkey.	Quantitative study using questionnaires	Self-administered questionnaire on definitions of euthanasia, legal aspects of euthanasia, expectations about euthanasia and attitude towards euthanasia.	25.5
Pelin and Arda, 2000, English/Turkey [74]	What are ethical attitudes of physicians in Ankara in 1995-1996?	524 **physicians.**	Quantitative study using questionnaires	Self-administered questionnaire on attitudes. e.g. towards informing patients about disease process and poor prognosis, alternative medicine, euthanasia.	15
Tepehan et al., 2009, English/Turkey [74]	Has working in ICUs an impact on attitude to euthanasia?	205 **physicians** and 206 **nurses** working in internal medicine, surgery and pediatrics in 3 hospitals.	Quantitative study using questionnaires	Self-administered questionnaire on definition of euthanasia, attitude to euthanasia, number of euthanasia requests and experiences and expectations about euthanasia.	34
Turla et al., 2006, English/Turkey [76]	What are attitudes of health professionals towards euthanasia?	545 **professionals** working at health clinics and hospitals.	Quantitative study using questionnaires	Self-administered questionnaire on definitions, legal status and ways of performing euthanasia, euthanasia requests, attitudes and expectations about euthanasia.	24.5
Van den Bosch, 2010, Dutch/Netherlands [77]	What is the quality of life of Turkish-Dutch elderly? What are experiences with health care and what factors influence confidence in GPs?	Interviews: 17 Turkish **elderly.** Questionnaire: 50 Turkish **elderly.**	Mixed-methods study combining interviews with questionnaire	Topics in interviews and-self administered questionnaire concerned experiences with ageing, communicating with GP, use of professional care and health condition.	28
Yaguchi et al., 2005, English/Turkey [79]	What are end-of-life attitudes of ICU physicians?	1961 **ICU physicians**, all participants of International Symposium on Intensive care and Emergency Medicine in Brussels.	Quantitative study using questionnaires	Self-administered questionnaire on treatment, use of do-not-resuscitate orders, strategy on mechanical ventilation and use of morphine in a constructed case.	27
Yerden, 2000, Dutch/Netherlands [80]	What are mutual expectations of Turkish elderly and their children with regard to traditional care? What care is given and what were consequences within family?	28 **elderly** and 32 **relatives** of 15 families: 10 active **elderly**, 8 in need of care and 10 bedridden.	Qualitative study using semi-structured interviews	Interview topics concerned migration experiences, education and work, social relations in Netherlands and in Turkey, living conditions, care of parents by children, professional care.	26
Yerden, 2004, Dutch/Netherlands [81]	What housing conditions can help care-dependent bedridden Turkish patients and their family members in combining family care with professional care?	16 **elderly** patients, 11 **partners** and 28 **children/grandchildren**: 8 elderly in need of care and 8 bedridden, 3 living in nursing home.	Qualitative study using semi- structured interviews	Interview topics concerned care needs and expectations, caring tasks, social networks, contacts between parents and children, contacts with neighbours, use of professional care, housing conditions, knowledge and use of modifications in the home and wishes about housing.	19
Yildrim et al., 2009, English/Turkey [83]	What is level of hopelessness among Turkish patients with cancer? How is this related to depression, anxiety and disease-related factors?	95 **patients.**	Quantitative study using questionnaires	Questionnaire based on questions of Pain Numeric Rating Scale, Beck Hopelessness Scale and Hospital Anxiety and Depression Scale.	34.5
*Findings concerning Moroccan patients*					
McCarthy et al., 2004, English/Morocco [63]	What are current practices of pain management among health professionals caring for children with cancer in Morocco? What are cultural and contextual influences for pain management?	14 **nurses** and 11 **physicians** caring for children with cancer.	Qualitative study using focus groups	Topics in the focus groups concerned pain assessment and management, training and resources, cultural influences and beliefs about pain management, need for assessment and treatment cancer-related pain in pediatric oncology.	32.5
Errihani et al., 2005, French/Morocco [52]	What are psycho-social characteristics of patients treated in Cancer Institute?	1000 **patients** with a histological confirmed cancer.	Quantitative study using questionnaires	Self-administered questionnaire on identity and origin, cultural and economic position and effects of illness on patient and family.	18
Errihani et al., 2006, English/Morocco [50]	What are psycho-social features of Moroccan breast-cancer women?	600 female **patients** with a histological confirmed breast cancer.	Quantitative study using questionnaires	Self-administered questionnaire on repercussion of disease on patient and family.	22.5
Errihani et al., 2008, English/Morocco [51]	What is impact of cancer on Moroccan patients of Moslim faith?	1600 **patients.**	Quantitative study using questionnaires	Self-administered questionnaire focusing on the repercussions of the disease on religious belief and practices.	19
*Findings concerning Turkish and Moroccan patients (sometimes including other immigrant patientsas well)*					
ACTIZ, 2009, Dutch/ Netherlands [22]	What factors are successful in providing intercultural care in home care and elderly care organizations?	44 **nursing professionals and relatives.**	Qualitative study using interviews, focus groups and literature	Topics were accessibility of formal care provisions, acceptance of discussing diseases, contact with family members, translation problems, religion and traditions, special care wishes and care provision.	22
Buiting et al., 2008; Buiting et al., 2009, English/Netherlands [35,36]	Are frequency and characteristics of end-of-life practices of non-Western migrants different from Dutch natives?	5342 **physician**s who signed death certificate for a non-sudden death.	Quantitative study using questionnaires	Self-administered questionnaire on withholding and withdrawing medical treatments, alleviation of pain or other symptoms or prescription of drugs taking into account or with explicit intention of hastening death.	36
De Graaff, 2002; De Graaff et al., 2005; de Graaff and Francke, 2003a; De Graaff and Francke, 2003b, English/Netherlands [17,42-44]	What experiences do Turkish and Moroccan families of terminally ill patients have of Dutch home care in terminal phase? What factors influence the access to and use of home care in the terminal phase?	**Relatives** of 9 Turkish and 10 Moroccan terminally ill patients.	Qualitative study using semi-structured interviews	Topics were the health situation of the patient in his last half year, how family care and eventually home care was organized, the care process, felt needs and how these needs were met.	29
De Graaff et al., 2005; De Graaff and Francke, 2009, English/Netherlands [44,45]	What experiences do GPs and home care nurses have with regard to home care for terminally ill Turkish and Moroccan migrants in the Netherlands? What factors influence their access to and use of home care?	88 **GPs** and 93 **nurses**, who had cared for terminally ill Turkish and Moroccan patients.	Quantitative study using questionnaires	Self-administered questionnaire on care for their last terminally ill patient, these patients’ needs and barriers to use of home care, their care giving and cooperation with other professionals.	28
De Graaff et al., 2010b; De Graaff et al., 2010a, English/Netherlands [18,46]	What do cancer patients with a Turkish or Moroccan background mean by ‘good palliative care? How do Dutch care providers deal with ideas that diverge from their own ideas about palliative care?	6 **patients**, 30 **relatives,** 17 **GPs**, 19 nurses, 5 **specialists**, 4 **social workers** and 2 **pastoral workers**, involved in palliative care for 19 Moroccan and 14 Turkish cancer patients.	Qualitative study using semi-structured interviews	Topics were decisions made on care and treatment in palliative stage, an evaluation of these decisions and one’s own view on communication in process.	34
Koppenol et al., 2006, Dutch/Netherlands [59]	What are perceptions of immigrant cancer patients on behavioural patterns, communication with care providers and use of health care?	Focus groups: Turkish, Moroccan, Surinamese and Caribbean **health advisors.**	Qualitative study using focus groups	Keywords concerned disease, immigrant groups, heath attitudes, communication, and health care utilization. Topics were cultural and religious aspects, social aspects, knowledge, personal emotions, informing patient of diagnosis and prognosis, informational needs, communication with care providers, use of treatments and care, psychosocial support.	21
Korstanje, 2008, Dutch/Netherlands [60]	What are needs and experiences of family members of immigrant patients in a Dutch hospital?	7 **relatives** of Turkish, Moroccan and West African Moslim immigrant patients.	Qualitative study using interviews	Keywords concerned family/informal care, intercultural care, immigrants, nursing, hospital. Topics were hygiene, religion, supporting patient, discharge from hospital, communication and becoming overburdened .	18
Meulenkamp et al., 2010, Dutch/Netherlands [65]	What are wishes of elderly immigrants and what support do they need to achieve quality of life?	83 **elderly** immigrants or their **relatives,** including 14 Turks and 12 Moroccans.	Qualitative study using interviews	Topics on physical welfare and health, living conditions, social participation and mental welfare.	35
Mostafa, 2009, Dutch/Netherlands [66]	What are experiences of immigrant women with breast cancer and what do they expect of their GP?	Interviews with 3 Afghan, 2 Moroccan, 2 Turkish and 1 Iranian **patients and** focus groups with **patients** and professionals (e.g. **nurses, physicians).**	Qualitative study using interviews and focus groups	Topics were: translation problems, communication, roles in care giving, cultural views, religion and personal experiences.	24
NOOM, 2009, Dutch/ Netherlands [23]	What is known among active senior immigrants about vulnerability of elder immigrants in Netherlands?	3 focus groups with **elderly** immigrants + Interviews with key **informants.**	Qualitative study using focus groups and interviews	Topics were body and mind, social relations, material conditions, work and productive life, values and inspiration.	18
Van Wijmen et al., 2010, English/Netherlands [78]	To what extent Dutch people know of existence of advance directives (ADs)? Which people do draw up an AD and for what reasons?	1402 **participants of a national panel** of consumers of health services, representative of Dutch population (also involving immigrants).	Quantitative study using questionnaires	Questions concerned ADs and end-of-life issues, such as reasons for possession of ADs, awareness of ADs, preferences, experiences and expectations concerning dying, end-of-life care, decision making and quality of life.	29
VPTZ, 2008a; VPTZ, 2008b, Dutch/Netherlands [25,26]	What are concerns of immigrants when caring for terminally ill?	140 Moroccan **relatives** in Rotterdam and 115 Turkish relatives in Enschede.	Qualitative study using focus groups	Topics were views on ‘good care’, best place to die, preferences on who is caring, allocation of tasks in family and in professionals etcetera.	21
Yerden and Van Koutrike, 2007, Dutch/ Netherlands [82]	What care is given by family members in immigrant families?	49 **relatives** (17 Turkish).	Qualitative study using focus groups	Topics were social networks, care duties, communication in family and use of professional care.	19

* Only research questions and characteristics of data collection related to the questions addressed in this systematic review are reported.

### Methodological quality

As said, the methodological quality of all the selected publications was assessed by one reviewer and half of the publications by a second reviewer as well. Scores from the first reviewer varied between 12 and 36 with a mean of 25 (see the Methods section for the range). Those from the second reviewer ranged from 16 to 36 with a mean of 27.4. The Pearson correlation between the scores of the two reviewers was 0.75 (p < 0.05). Cohen’s kappa for the between the category scores was also significant (p < 0.05).

The total score for each study is presented in Table 5 (the mean score is presented for studies assessed by both reviewers). Six studies fell in the ‘low methodological quality’ category, 32 in the ‘moderate’ category and 19 in the ‘good quality’ category. The aspects most frequently given a low score were ‘background and clear statement of the research aims’, ‘addressing ethical issues’ and ‘sampling strategy’. The six studies [23,28,37,52,60,74] with low scores did not show results that were fundamentally different or contradictory to the other results and are therefore not excluded or treated differently in the synthesis.

We felt that the findings from our study could best be presented by clustering them into four themes, namely the experiences and perceptions regarding family care, those regarding professional care, those regarding end-of life care and decision making, and finally the experiences and perceptions regarding communication in end-of-life care.

### Findings related to perspectives regarding family care

Seventeen studies addressed the contribution of family members in the care for incurable Turkish or Moroccan patients. Three topics were mentioned frequently: family care is seen as (1) a social duty, (2) an economic necessity, and (3) a burden.

#### Family care as a duty

All studies – whether they were performed in the countries of origin or in the host immigrant countries - indicate that family members are key care providers. For example, it was shown by studies conducted in Turkey that family care is a duty [28], and that Turkish patients are mainly cared for by the family [68]. In addition, studies performed in Morocco showed that most patients (92%) were supported by the family [50,52]. Studies performed in the Netherlands also stress that Turkish and/or Moroccan patients and their relatives consider family care as an obvious duty [17,55,80,81]. Many patients expected their children and their children’s partners to take care of them. In practice daughters or daughters-in-law did most of the work [17,55,81,82]. Family care involved activities like preparing food all through the day for the patient and for the many visitors, doing the paperwork, accompanying the patient to the doctor as an interpreter and providing personal care [55,65,81].

#### Family care as an economic necessity

Some studies – performed in the home countries - noted that the emphasis on family care is also the result of poverty. In Turkey, families’ poverty and hospitals’ lack of resources were seen as problem by physicians [40]. In Morocco, cancer patients said that professional treatment was frequently compromised by poverty and lack of medical insurance [50,52]. Often families had insufficient monthly income to pay for professional medical treatment.

#### Family care as a burden

Family care is considered burdensome - especially in the Dutch studies. Studies conducted among Turkish families living in the Netherlands indicated that sometimes sons and daughters of Turkish patients decided to move out in order to flee from the duty they saw as too heavy [80]. One reason for relatives’ exhaustion was the contrasting opinions of patients and relatives regarding the feasibility of family care [17,81]. Other studies found that relatives felt that the illness imposed a heavy financial burden on the family, as the health-care insurance did not cover all the costs of family care [26,60,77].

### Findings relating to perspectives regarding professional care

Twenty-five studies addressed the contribution of professionals in the care for incurably ill Turkish and Moroccan patients. Three topics were mentioned frequently: (1) a preference for hospital care, (2) barriers to professional care and (3) the quality of professional care.

#### A preference for hospital care

In Turkey it was found that 43% of the gynaecological cancer patients (in cases where cure was no longer an option) preferred to stay in the hospital, 41% preferred outpatient care and 16% wished to go home; the main reason for wanting to stay in hospital was the feeling of security [32]. Turkish professionals were more likely to want terminal patients to leave the hospital as they accepted that hospitals cannot cure these patients [28,64]. Ersoy and Gundogmus found that “most GPs preferred to hospitalize the patient, even by using force if necessary, in order to keep the patient from harm”. Turkish physicians were more inclined to fulfil the wishes of the patients’ family than to respect the patients’ wishes [54]. In a study performed in the Netherlands, most Turkish and Moroccan patients preferred to die at home, but being in the hospital was preferred if they still hoped for a cure or wanted to relieve the family [26]. Buiting et al. found that immigrants were more likely to die in hospital than Dutch patients [35].

#### Barriers to the use of professional care

According to patients, relatives and professionals, the main reasons for immigrants’ limited use of professional home care, residential care for the elderly or hospice care were unfamiliarity with the available care facilities and language barriers [33,45,60,77]. Also financial problems and traditional views on family duties sometimes formed barriers to using professional care [17,23,26,80]. Relatives’ care preferences and feelings of shame were often a deciding factor in the limited use of professional care [17,47,80].

#### Perspectives on the quality of professional care

The quality of care was discussed mainly from the perspective of professionals. It was found that the use of hospital care in Turkey was hampered by limited communication between patient and relatives, and limited cooperation among professionals [64]. Another study reported that more than half of the nurses working in an oncology centre in Ankara had experienced inadequate pain management [67]. Furthermore, nurses and physicians working in oncology centres in Morocco felt embarrassed by the lack of resources and limited training in the treatment of cancer-related pain [63].

In the Netherlands, relatives of patients with a Turkish or Moroccan background often felt responsible for the care being given, making them very critical of the professionals’ activities [22,60,65]. Professionals in the Netherlands felt the insufficient quality of palliative care for immigrant patients was mainly due to communication problems [18,23,45,60].

### Findings relating to perspectives regarding end-of-life care and decision making

Thirty-five studies addressed the perspectives regarding end-of-life care or decisions at the end-of-life. Five topics were mentioned frequently: (1) hope and faith, (2) views regarding euthanasia, (3) withdrawing and withholding treatment, (4) artificial nutrition and continuing to offer food, and (5) involvement in decision-making.

#### Hope for cure and faith in Allah

Many studies revealed that Turkish and Moroccan patients often strive for maximum treatment right up to the end of life. For instance, 63% of the gynaecological cancer patients in the study by Beji et al. (2005) asked for life-sustaining treatments [32]. Patients only refused life-sustaining treatments in cases where the patient was suffering from poor family relationships, pain or depression. Errihani et al. (2008) found that patients with cancer who were not practicing Muslims (49%) often felt guilty, while active believers commonly accepted cancer as a divine test [51].

In the Netherlands, the keenness among Turkish and Moroccan patients to have life-sustaining treatments was confirmed in a study by De Graaff et al. [18]. Dutch physicians noted that immigrants were more likely than Dutch patients to be offered life-prolonging treatments (20% vs 12%), artificial respiration (38% vs 16%) and cardiovascular medication (30% vs 11%) [35]. Some families related their wish for life-prolonging treatments to their Muslim religion [18,22,59,60]. Some relatives also mentioned the wish to let the patient die with a clear mind, enabling a good start in the hereafter [18].

#### Perspectives on euthanasia

In the Netherlands euthanasia is defined as being the termination of life by a doctor at the request of a patient [3]. This also includes physician-assisted suicide. Euthanasia is not taken to mean abandoning treatment if (further) treatment is pointless. In such cases it is considered part and parcel of normal medical practice that the doctor discontinues treatment and lets nature take its course. The same applies to administering large doses of opiates for pain relief whereby one side effect is that death occurs more quickly.

Several studies in Turkey addressed the concept of ‘euthanasia’, although what respondents meant by this concept varied. One study recorded that Turkish nurses and doctors did not have the same knowledge about the different forms of euthanasia (active euthanasia, passive euthanasia, physician-assisted suicide and involuntary euthanasia) [75]. They were most familiar with passive euthanasia (73.2% of doctors and 62.6% of nurses). In a study by Oz, most nurses and physicians (58%) defined it as “allowing death, leaving patients to die”, others (17%) defined it as “passive euthanasia, not active death determined by others”, or as “painless, peaceful death” (13%) [69].

Some Turkish studies suggest that professionals do sometimes get requests to perform euthanasia or “to make death easy”. The number of professionals that had been asked to do so varied (see Table 3), ranging from 8% of the professionals in the study by Turla et al. [76] to 34% of the oncologists in the study by Mayda et al. [62]. The different percentages may be related to the above-mentioned differences and/or a lack of clarity in definitions of euthanasia or “making death easy”. The responses to a patient’s request for euthanasia or “making death easy” also varied (see Table 3). Most professionals in Turkey were disapproving of any form of legalization of euthanasia. The reasons for objecting to legalization were fear of abuse, ethical principles, religious beliefs and personal values [62,75,76].

No Moroccan studies about views on euthanasia were found. But in the Netherlands, a study concluded that patients and families with a Dutch background were more likely to request euthanasia (25% and 18%) than migrants and their relatives (3% and 4%) [35].

#### Withdrawing or withholding life-prolonging treatments

One study found that older Turkish patients often opted for life-prolonging treatments; even when they longed to die, they accepted life-prolonging proposals from physicians as their relatives wanted them to live as long as possible [31]. Another study noted that 38% of the Turkish physicians had advised patients not to start life-prolonging therapy and 51% had withdrawn treatment [62]. Although withdrawing treatment was often felt to be reprehensible, professionals also admitted it to be part of their job. In one study, for example, 40% of the Turkish intensive-care nurses justified withdrawing treatment when there was no medical benefit [27], and another study [75] found that 40% of the intensive-care physicians had discontinued treatment in patients with an incurable disease on more than one occasion. Withdrawing treatment was more difficult than not initiating the treatment [40], and was often a matter for discussion. 84% of the Turkish physicians in the study of Ersoy and Gundogmus found withholding treatment acceptable if the patient wished for it, but only 13% would respect the patients’ wishes if relatives contested this [54]. Ilkilic described how Turkish parents living in Germany wished to continue mechanical respiration of their incurably ill child, referring to their religious duties, while the German physicians judged the situation to be medically hopeless [56].

#### Continuing to offer food and artificial nutrition

Several studies reported that Turkish patients often expect to be fed until the very end. In one study 75% of health staff disagreed with the statement that, nutrition should be stopped if a patient wants euthanasia [58], while 68% of the nurses in another study agreed that artificial nutrition should always be continued [27].

No Moroccan studies were found on this topic, but in the Netherlands it was reported that Turkish and Moroccan immigrant families preferred the feeding of their terminally ill relatives to continue [18]. Besides, it was found that terminally ill immigrants in the Netherlands were more likely to be given artificial nutrition and hydration than comparable native Dutch patients [35].

#### Involvement in end-of-life decisions

In Turkey, many cancer patients wanted to be involved in decisions about treatments (79% of the sample in one study, for example [49]), but use of written advanced directives was low and do-not-resuscitate orders were often only given verbally [57,79]. In practice, relatives were often the ones making the decision in end-of life care. In one study, 54% of the Turkish oncologists said patients should decide about euthanasia and 42% said families and doctors should decide jointly [62]. The strong involvement of relatives in decision making is also described by Iyilikçi et al. [57]. However, some studies also pointed to preferences for taking decisions jointly: Iyilikçi et al., for example, concluded that most anaesthesiologists in Turkey wanted decision by consensus [57]. Moreover, Turla et al. reported that 63% of the professional care providers wished that “both the physician and the family” could decide [76]. Yet other authors remarked that decisions were often still the domain of physicians [31,49,71,74].

This topic was not addressed in studies performed in Morocco, but Dutch research indicated that Turkish and Moroccan relatives sometimes did not want any medical end-of-life decisions to be taken as the end of life ought to be in the hands of Allah [22]. In other studies relatives declared that they should be the main party in the decision-making and not the patient, as the latter deserved rest and had to remain hopeful until the end [18,23]. However, this contrasted with the dominant view of Dutch professionals that decision making should always reflect the preferences of the individual patient.

### Findings relating to communication

Thirty-seven studies addressed the communication between Turkish and Moroccan patients and relatives and their care providers in end-of-life care. Four topics were mentioned frequently: (1) communication about diagnosis and prognosis, (2) communication about pain, sorrow and mental problems, (3) language barriers and (4) communication patterns within the family.

#### Communication about diagnosis and prognosis

Most studies on communication about diagnosis and prognosis were performed in Turkey. In this country the percentages of patients unaware of their diagnosis varied (see Table 4), ranging from 16% [48] to 63% [37]. The wish to be informed varied from 66% of the patients with diverse diagnoses [48] to 85% of patients diagnosed with cancer [31,49]. Relatives may form barriers to informing patients about a bad diagnosis or prognosis. In one study, it was found that many relatives (66%) did not want patients to be informed because they would be upset or would not want to know it [71]. Another study found that 39% of the relatives adhered to this opinion, 48% felt that patients should be informed, and 13% were hesitant [68]. The differences between the findings might be due to the differences in the formulation of the questions.

The likelihood of Turkish physicians informing patients increased with an increase in the patient’s socio-economic status and educational level [37], and also depended on the type of illness and on relatives’ preferences [22,54]. Although 93% of the physicians in the study by Pelin and Arda [74] thought that patients should be informed, 30% chose to inform relatives. While 67% of the physicians in another study would tell the truth to the patient, 8% preferred to inform relatives and 12% would ask relatives for their consent before talking to the patient [54]. However, 68% of the physicians in a third study said they would tell the patient the diagnosis first before informing their relatives [31]. Professionals’ attitudes were influenced by their skill in bringing bad news and by the stage of the disease. Trained and more experienced physicians were more likely to inform the patient [72,74]. Furthermore, 76% of nurses would tell the truth to a breast-cancer patient asking for a diagnosis [53], while 96% would not inform a patient in the terminal phase [67].

In Morocco, 33% of cancer patients did not know their diagnosis, while relatives were informed in 89% of cases [52]. In the Netherlands, some Turkish and Moroccan patients were not informed [17,33,45,59]. Elderly patients would not talk about life-threatening illnesses [26], whereas younger patients often preferred to be informed but would not inform all their relatives [59]. The arguments used were that telling the truth would hasten a patients’ death and that information might stir gossiping in the community [56,59]. In addition, Turkish and Moroccan relatives disliked the direct way Dutch care providers informed patients [18,23,26]. The influence of relatives in communication is amplified by their role as interpreters [18,33,59].

#### Communication about pain, sorrow and mental problems

Several studies noted that communication with a patient at the end of life is often problematic. For example, one study found that Turkish patients often did not want to talk about pain as they feared becoming dependent on analgesics and did not want to upset their relatives [30]. Atesci et al. found more psychiatric disorders among patients aware of their diagnosis than among patients who were not informed, but this might be related to inadequate information so the authors concluded that physicians should teach patients to cope with the information [29]. Turkish nurses felt embarrassed because they could not express their feelings of empathy for dying people [64].

McCarthy et al. reported that Moroccan physicians and nurses did not have the means to detect or assess pain as their patients did not indicate pain, either physically or verbally [63]. Studies in the Netherlands report that, according to patients and relatives, immigrant patients would often not talk about psychological problems, depression or dementia [23,66,77].

#### Language barriers

In a Moroccan study it was noted that 25% of the patients spoke only Berber. Their insufficient knowledge of Arabic caused enormous difficulties in communication between patients and professionals [50].

According to patients, relatives and care providers in the Netherlands, language barriers were often tackled with the help of relatives and bilingual interpreters or other intermediary professionals [17,18,60]. Many elderly Turkish patients living in the Netherlands said they wanted Turkish-speaking staff [33]. This was sometimes arranged [77]. But involving staff of Turkish or Moroccan origin could also amplify undesirable social control within the community [26,59]. In another immigrant country, namely Germany, it was noted that language barriers impeded physicians in taking joint decisions with Turkish patients - they could not understand the discussions between patients and interfering relatives [56].

#### Communication within the family and within the community

Many studies suggested that communication problems in palliative care were sometimes related to the social patterns within the family. For example, one study concluded that according to patients, the dominance of families in patient support often resulted in low disclosure rates in Turkey [34]. In a study performed among Turkish and Moroccan health advisors in the Netherlands, it was found that Turkish and Moroccan immigrant families seldom talk about illness and the sorrow it causes because they wanted to avoid gossiping in the community [59]. According to elderly immigrants, immigrant families were facing a dilemma: the ideal of children taking care of parents prevented them from discussing care needs openly and from looking for other sources of care [23]. The explanations given for limited communication within the family were immigrants’ limited experience with dying, as the previous generation was cared for in their country of origin [18], a lack of knowledge about the facilities in the host country [26] and religious traditions: the obligation to provide care, and pressure from the community, be it Muslims [17,80] or Christians [55].

## Discussion

This systematic literature study describes the care experiences and care perceptions of incurably ill Turkish and Moroccan patients, their relatives and care professionals, and their communication with each other.

The extensive searches resulted in 64 relevant references dealing with 57 studies. Eighteen of the 57 studies concerned *family care.* These studies showed that relatives considered family care as a duty, although the care burden was often too high for the female relatives in particular. This conclusion was mainly based on studies of Turkish and Moroccan immigrant families living in the Netherlands as family care has never been a central issue in the studies in the countries of origin (Turkey and Morocco).

Twenty-five studies addressed *perceptions regarding professional care.* A lot of Turkish and Moroccan incurably ill patients and their relatives preferred hospitalization, which was related to their search for security and a cure right up to the end. In addition, the limited use of professional home care and residential elderly care was also due to financial problems, preferences for family care, communication problems and the insufficient quality of care. However, it should be noted that the preferences for certain kinds of care will depend on the availability of alternatives. This could explain why preferences for hospital care were addressed mainly in Turkish studies, while the thresholds to using professional home care, residential care for the elderly and hospice care were mainly studied among immigrants in the Netherlands.

On the basis of 35 studies addressing the perspectives concerning *end-of-life care and decision making,* we can conclude that patients and family often wanted life-prolonging treatments until the very end. Decisions to withdraw or withhold treatments were often contested by relatives and not openly discussed with the patient. Hope for a cure and the desire for life-prolonging treatments were related to faith in Allah. Patients’ and relatives’ focus on life prolongation might be a reason for the fact that perceptions regarding euthanasia and views on withdrawing or withholding treatments were only investigated in studies among professionals. These studies – mainly performed in Turkey - showed that, in general, withdrawing treatment and euthanasia were disapproved of. But the findings of these studies were not congruent in all regards: some studies emphasized a preference for shared decision-making, while other studies noted that decisions were still often made by physicians or relatives, with the patients’ opinion not being taken into account.

Thirty-seven studies addressed *communication.* Incurably ill patients with a Turkish or Moroccan background were not always informed about their diagnosis although professionals frequently held the opinion that patients should be informed. Relatives often prevented disclosure as they felt this might upset their patient, even when patients wished to know the truth. Additionally, communication about pain and psychological symptoms was often problematic because patients were reluctant to talk, fearing the use of analgesics and wanting to avoid upsetting their relatives. Language barriers and the dominance of the family reinforced the often complex communication patterns. Communication was often hampered by language barriers (in the Netherlands, but also in Morocco) or the relatives’ dominant role.

A limitation of this review is that we could not synthesize the data of underlying studies in all regards because of the variety of instruments and research questions. Findings were frequently not in agreement, even on topics that have been studied rather intensively (such as informingpatients about the diagnosis and prognosis), due to diverging research questions, designs and instruments.

Account must also be taken of the fact that the representativeness of the results is sometimes open to discussion, as the response rates in the quantitative studies were often unknown or low, and the qualitative studies had small samples.

Another limitation is that the majority of the studies included in the review concerned professionals in Turkey or immigrants’ relatives in the Netherlands. In general the patient perspective was under-represented, and there were only a limited number of studies in Morocco, hampering comparisons between Turkish and Moroccan subjects. In addition, we cannot draw conclusions on the basis of this review as to whether the specific experiences and perceptions of Turkish and Moroccan immigrants are rooted in either their past in the country of origin or in their present situation as immigrants in a foreign country. Time may be an important factor, both for Turkish, Moroccan and Dutch professionals and for Turkish and Moroccan patients and their relatives. As VPTZ [26] indicated, a culturally sensitive approach may lead to an acceptance of the do-not-tell wishes of some elderly first-generation immigrants, but will not suit the information needs of second-generation immigrants.

This review confirms the view that palliative care should take account of the specific cultural characteristics of patients and relatives. In the Introduction section we referred to studies indicating that poverty, family systems, religious customs and traditional care influenced the care perceptions of patients in non-Western low-resource countries. This corresponds with our findings on incurably ill patients with a Turkish and Moroccan background. In addition, we referred to studies showing that palliative care for immigrants in Western countries is often hindered by language barriers and health illiteracy, resulting in an important role for interpreting relatives. Comparable communication features were found in this review dealing specifically with people with a Turkish or Moroccan background.

A question that subsequently arises is whether our findings on Turkish and Moroccan patients and families can also be applied to other immigrant groups in the Netherlands, such as Moluccan, Chinese, Surinamese and Antillean immigrants, and refugees from various countries. No unequivocal conclusions can be drawn on the basis of this review as the immigrant groups mentioned differ from Turkish and Moroccan immigrants in terms of of the countries they come from, when and why they immigrated to the Netherlands, their reception in the Netherlands and Dutch language skills. Future research on care beliefs, needs and communication processes in the palliative care phase for other immigrant groups in the Netherlands could bolster and extend the current insights.

## Conclusions

This systematic literature study confirms the findings of our earlier empirical studies, showing that family care for incurably ill patients is considered a duty, even when this care becomes a severe burden for the central female family caregiver in particular. Hospitalization is preferred by a substantial proportion of patients and relatives, since they often strive for cure right up to the end and focus strongly on life-prolonging treatments. Relatives often prevent disclosure as they feel this might upset their patient, even when patients wish to know the truth. Medical end-of life decisions, such as withdrawing and withholding treatment, are seldom discussed with the patient. In addition, communication about pain or mental issues is limited. Language barriers and the dominance of the family may amplify communication problems. Apart from these concrete findings our review revealed that the perspectives on care and communication involving incurably ill Turkish and Moroccan patients not only reflect their cultural background but also their social situation. This dual focus enables palliative care policy makers and practitioners to take account of the social and economic circumstances of each family, the mental and physical barriers preventing them from engaging professional care, the religious and legal considerations influencing their decision making and their communication capacities.

## Competing interests

The authors declare that they have no competing interests.

## Authors' contributions

FMG and ALF designed the study; FMG and PM performed all phases of the systematic review; WD and ALF were involved in the methodological assessment; FMG wrote initial draft of this paper and PM, WD and ALF gave comments on all following versions and the final version. All authors read and approved the final manuscript.

## What is already known about the topic?

The international literature has shown that palliative care for non-Western patients can be problematic because of inadequate pain relief and medication, as wells as family systems and religious practices. In addition, palliative care for ethnic minorities in Western countries can be limited by languages barriers, ignorance about specified facilities, discrimination and criticism from their community.

## What the paper adds

Our review looking at Turkish and Moroccan patients demonstrates that the ‘family’ factor is crucial. Palliative care for Turkish and Moroccan patients has to take account of the fact that family care is dominant and that the desire for a cure leads to a preference for hospital care, even when cure is no longer an option. Efforts to inform incurably ill patients about their diagnosis and prognosis, and share decisions regarding medical end-of life decisions are often contested by relatives, who want to protect the patients and keep hope alive. Even when patients want to know the truth, they are often protected by their relatives.

## Pre-publication history

The pre-publication history for this paper can be accessed here:

http://www.biomedcentral.com/1472-684X/11/17/prepub

## Supplementary Material

Additional file 1 Search sources. Click here for file
