# Supplementary material for: Perspectives on care and communication involving incurably ill Turkish and Moroccan patients, relatives and professionals: a systematic literature review
Source: BMC Palliat Care. 2012 Sep 18;11:17. doi: 10.1186/1472-684X-11-17 (PMC3517329; doi:10.1186/1472-684X-11-17)
Supplement: Additional file 1 — Search sources. [file 1472-684X-11-17-S1.doc]

**Additional file 1 - Search sources**

|  | **Literature Databases** | **Specific journals** | **Websites** |
| --- | --- | --- | --- |
| 1 | Pubmed | [Journal for Medical Antropology] (Dutch) | [www.forum.nl](http://www.forum.nl/) |
| 2 | Embase | [Culture Migration and Health] (Dutch) | [www.nigz.nl](http://www.nigz.nl/) |
| 3 | Psychinfo | [Dutch Journal for palliative care] (Dutch) | [www.vilans.nl](http://www.vilans.nl/) |
| 4 | Cinahl | [Pallium] (Dutch) | [www.actiz.nl](http://www.actiz.nl/) / [www.actizkleurrijkezorg.nl](http://www.actizkleurrijkezorg.nl/) |
| 5 | Lilacs  (Latin American and Caribbean Health Sciences Literature) |  | [www.kit.nl](http://www.kit.nl/) |
| 6 | IMEMR  (Index Medicus for the WHO Eastern Mediterranean Region) |  | [www.mikadonet.nl](http://www.mikadonet.nl/) |
| 7 | Science Citation Index |  | [www.stichtingak.nl](http://www.stichtingak.nl/) |
| 8 | Sociological abstracts |  | [www.netwerknoom.nl](http://www.netwerknoom.nl/) |
| 9 | Cochrane library |  | [www.kwfkankerbestrijding.nl](http://www.kwfkankerbestrijding.nl/) |
| 10 | Campbell library |  | [www.venvn.nl](http://www.venvn.nl/) |
| 11 | Scirus |  | [www.sting.nl](http://www.sting.nl/) |
| 12 | ATLA religion database |  | [www.palliatief.nl](http://www.palliatief.nl/) |
| 13 | Anthrosource |  | [www.vptz.nl](http://www.vptz.nl/) |
| 14 | Abstracts in Anthropology |  | [www.nivel.nl](http://www.nivel.nl/) |
| 15 | Global health |  | [www.vikc.nl](http://www.vikc.nl/) |
| 16 | Bibliography of the Dutch Caribbean |  | [www.pacemaker.nl](http://www.pacemaker.nl/) |
| 17 | Picarta  (Dutch manuscripts & books) |  | [www.verwey-jonker.nl](http://www.verwey-jonker.nl/) |
| 18 |  |  | [www.rouwrituelen.nl](http://www.rouwrituelen.nl/) |
| 19 |  |  | [www.interculturelecommunicatie.com](http://www.interculturelecommunicatie.com/) |
| 20 |  |  | [www.alleato.nl](http://www.alleato.nl/) |
| 21 |  |  | [www.pharos.nl](http://www.pharos.nl/) |
| 22 |  |  | [www.zonmw.nl](http://www.zonmw.nl/) |
| 23 |  |  | [www.imes.uva.nl](http://www.imes.uva.nl/) |
| 24 |  |  | <http://epoc.cochrane.org/specialised-register> |
| 25 |  |  | [www.mighealth.net](http://www.mighealth.net/) |
| 26 |  |  | [www.eniec.eu](http://www.eniec.eu/) |
| 27 |  |  | [http://europa.eu](http://europa.eu/) |
| 28 |  |  | [www.who.int](http://www.who.int/) |
| 29 |  |  | <http://www.icmh.ch/> |
| 30 |  |  | <http://iccnetwork.org/> |
| 31 |  |  | <http://www.tcns.org/> |
| 32 |  |  | [www.xculture.org](http://www.xculture.org/) |
| 33 |  |  | <http://www.omhrc.gov/> |
| 34 |  |  | <http://www.med.umich.edu/multicultural/index.html> |
| 35 |  |  | [www.ahrq.gov](http://www.ahrq.gov/) |
| 36 |  |  | [www.amazon.com](http://www.amazon.com/) |
| 37 |  |  | [www.books.google.com](http://www.books.google.com/) |
